# Association of FLAIR Positivity and Worse Outcomes After Intravenous Thrombolysis in Known-Onset Strokes: A Systematic Review and Meta-Analysis

**DOI:** 10.3390/jcm14228031

**Published:** 2025-11-12

**Authors:** Esra Zhubi, Azamat Bissenov, Anna Sára Lengyel, Réka Tóth, András Attila Horváth, Szabolcs Kéri, Marie Anne Engh, Péter Hegyi, Bence Gunda

**Affiliations:** 1 Department of Neurology, Semmelweis University, 1083 Budapest, Hungary; 2 Centre for Translational Medicine, Semmelweis University, 1085 Budapest, Hungary; 3 Department of Orthopedics, Semmelweis University, 1083 Budapest, Hungary; 4 Neurocognitive Research Center, Nyírő Gyula National Institute of Psychiatry and Addictology, 1135 Budapest, Hungary; 5 Research Centre for Natural Sciences, HUN-REN, 1519 Budapest, Hungary; 6 Department of Anatomy, Histology and Embryology, Semmelweis University, 1094 Budapest, Hungary; 7 Sztárai Institute, University of Tokaj, 3944 Sárospatak, Hungary; 8 Department of Physiology, Albert Szent-Györgyi Medical School, University of Szeged, 6720 Szeged, Hungary; 9 Institute for Translational Medicine, Medical School, University of Pécs, 7624 Pécs, Hungary; 10 Institute of Pancreatic Diseases, Semmelweis University, 1083 Budapest, Hungary

**Keywords:** acute ischemic stroke, FLAIR, hemorrhagic transformation, intravenous thrombolysis, MRI

## Abstract

**Background/Objectives**: MRI FLAIR signal serves as a time surrogate in unknown onset strokes; however, its prognostic value in early known-onset strokes treated with intravenous thrombolysis (IVT) remains unclear. We aim to evaluate whether FLAIR positivity predicts hemorrhagic transformation and less favorable functional outcome following IVT within 4.5 h of symptom onset. **Methods**: A systematic search of Embase, PubMed, and Cochrane Library was conducted. We included studies reporting 90-day functional outcome and hemorrhagic transformation after intravenous thrombolysis within 4.5 h of symptom onset in relation to the FLAIR signal on pre-treatment MRI. Two reviewers independently extracted data and assessed bias using QUIPS. A random-effects model was used for meta-analysis. The study protocol was registered in PROSPERO (CRD42023486781). **Results**: Six studies comprising 951 patients were included in the final analysis. Patients with FLAIR-positive lesions (25.86%) had non-significantly higher odds of hemorrhagic transformation (OR, 3.47; 95% CI, 0.51–23.57; *p* = 0.131) and significantly higher odds of unfavorable 90-day outcome (OR, 2.14; 95% CI, 1.01–4.55; *p* = 0.049). The pooled sensitivity and specificity of FLAIR status for predicting hemorrhagic transformation were 41.2% and 82.1%. The pooled sensitivity and specificity of FLAIR status for predicting less favorable outcome were 40.9% and 78.1%, respectively. **Conclusions**: FLAIR positivity is significantly associated with less favorable 90-day functional outcome and a trend toward a higher rate of hemorrhagic transformation following IVT within 4.5 h of symptom onset. It may aid risk stratification but should not preclude treatment in eligible patients.

## 1. Introduction

Stroke represents one of the leading causes of death and a major contributor to disability globally [1]. Intravenous thrombolysis (IVT) remains a key treatment for acute ischemic stroke and the prevention of disability [2]. In recent times, the utilization of tissue plasminogen activator (tPA) has seen a steady rise, likely due to the extended time window for administration (>4.5 h after symptom onset), widespread awareness campaigns [3], and improved access to specialized treatment and imaging [4].

Although current guidelines recommend administering tPA intravenously to acute ischemic stroke patients within 4.5 h of symptom onset [2,5], there remains significant concern about the increased risk of hemorrhagic transformation and associated neurological deterioration, even among patients falling within this treatment timeframe [6]. IVT has been associated with a 6–8% risk of symptomatic intracranial hemorrhage within the standard treatment window [7], and up to a 34% risk of all types of hemorrhagic transformation [8]. Prior research has pinpointed several factors that increase the likelihood of hemorrhagic transformation and neurological deterioration following acute ischemic stroke [9,10]. These include age, smoking, hypertension, diabetes, high cholesterol, atrial fibrillation, severity of stroke, prior history of stroke, lesion size on diffusion-weighted imaging (DWI), and age-related alterations in white matter observed on magnetic resonance imaging (MRI) [11].

In unknown-onset strokes MRI fluid-attenuated inversion recovery (FLAIR) sequences, which do not contain focal hyperintensities in regions of diffusion restriction (FLAIR-negativity), serve as a reliable marker for detecting stroke occurring within 4.5 h of symptom onset amenable to thrombolysis [12,13]. However, FLAIR-positive lesions are often seen in known-onset strokes within the standard treatment time window and possibly reflect deeper ischemia and vasogenic edema already developed due to impairment of the blood–brain barrier [14]. Patients with FLAIR-positive strokes have been observed to have worse functional outcomes than patients with FLAIR-negative strokes and higher rates of disability and mortality [15]. Despite current research, it remains uncertain whether FLAIR status plays a significant role in predicting clinical outcomes following thrombolysis treatment, including functional recovery and the risk of hemorrhagic transformation [16,17,18].

This study aims to evaluate whether FLAIR positivity is associated with worse outcomes after IVT in known-onset strokes. We hypothesized that patients with acute ischemic stroke undergoing IVT within 4.5 h of known onset, who have a visible lesion on pre-treatment FLAIR, are less likely to have favorable functional outcome at 90 days. Moreover, we hypothesized that the visibility of lesions on FLAIR may be associated with an increased risk of hemorrhagic transformation after IVT.

## 2. Materials and Methods

### 2.1. Study Design

This systematic review and meta-analysis followed the PRISMA 2020 guidelines [19] and the Cochrane Handbook [20]. The study protocol was registered with PROSPERO (registration number: CRD42023486781) and was strictly followed throughout the research process. No ethical approval was required for this systematic review with meta-analysis, as all data was already published in peer-reviewed journals.

### 2.2. Information Sources and Search Strategy

The comprehensive search was performed in three databases—Medline (via PubMed), Embase, and the Cochrane Central Register of Controlled Trials (Central)—on 27 November 2023. Citationchaser [21] was utilized to locate relevant studies from the reference lists of the selected articles and the articles that cited them.

### 2.3. Eligibility Criteria

This analysis followed the patient, intervention, comparative intervention, and outcome (PICO) framework, focusing on comparing outcomes of IVT within the standard treatment window in patients with FLAIR-positive and FLAIR-negative acute ischemic lesions. The primary outcomes of interest were hemorrhagic transformation following IVT and functional outcome at 90 days, assessed using the modified Rankin Scale (mRS).

FLAIR-positive lesions were defined as acute ischemic lesions visible on DWI with a corresponding hyperintense signal on FLAIR, as visually identified by physicians. FLAIR-negative lesions were defined as acute ischemic lesions visible on DWI without a corresponding FLAIR signal. The inclusion criteria were (a) studies reporting on patients with known-onset ischemic stroke; (b) patients who received intravenous thrombolysis within the standard time window (≤4.5 h); (c) availability of pre-treatment brain MRI including DWI and FLAIR sequences; (d) availability of 24 h follow-up imaging (MRI or CT) after IVT; and (e) studies that provided outcome data on hemorrhagic transformation and/or 90-day functional outcome.

### 2.4. Search Strategy

We searched for domains of stroke, thrombolytic therapy, MRI and FLAIR. The following search key was used without applying any filters:

(“stroke” OR (“cerebral” AND “infarction”) OR (“brain” AND “infarction”)) AND ((“intravenous” AND “thrombolysis”) OR “IVT” OR (“thrombolytic” AND “therapy”) OR (“tissue” AND “plasminogen” AND “activator”) OR “rTPA” OR “alteplase” OR (“intravenous” AND “alteplase”)) AND ((“magnetic” AND “resonance”) OR “MRI” OR “fluid-attenuated inversion recovery” OR “FLAIR” OR (“DWI” AND “FLAIR” AND “mismatch”)).

No restrictions were imposed on the type of publications to be included, nor was the search limited by language or other criteria.

### 2.5. Selection Process

The articles obtained from our search query were imported into reference management software, specifically EndNote v20, by Clarivate Analytics based in Philadelphia, PA, USA, and Rayyan QCRI (https://new.rayyan.ai/, accessed on 27 February 2024) [22]. Duplicate entries were removed both automatically and manually using EndNote v20. Following this, two independent reviewers (EZ, AB) screened the articles by title, abstract, and full text using Rayyan QCRI. Any disagreements between the reviewers were resolved by consensus or by a third reviewer (AL). Inter-rater reliability was measured with Cohen’s kappa coefficient at each phase for inclusion in the study when individual decisions needed to be compared [23].

### 2.6. Data Collection Form

Data extraction from eligible articles was independently carried out by two authors (EZ, AB) using a data collection form in Microsoft Excel (Microsoft Corp. Microsoft Excel 2019, Redmond, WA, USA) [24]. The extracted data included the name of the first author, year of publication, basic demographic characteristics (number of patients, number of females, age), number of patients in FLAIR positive and negative groups, number of events in each group, length of follow-up period, and the outcome of interest, when available. Any discrepancies or inquiries during the data extraction process were resolved through discussions between the authors.

### 2.7. Study Risk of Bias Assessment

Two reviewers (EZ and AB) autonomously assessed the risk of bias in the studies utilizing the Quality In Prognosis Studies (QUIPS) tool [25]. Any discrepancies were resolved by a third investigator (AL).

### 2.8. Certainty of Evidence

We employed the Grading of Recommendations Assessment, Development, and Evaluation (GRADE) approach [26] to assess the quality of evidence for the outcomes in our clinical inquiry. GRADEpro GDT 2015 (software; McMaster University and Evidence Prime (Hamilton, ON, Canada), 2022, accessible from gradepro.org) was utilized to interpret the findings.

### 2.9. Statistical Synthesis

Given the considerable between-study heterogeneity presumed, random-effects models were employed to combine effect sizes in our meta-analysis. The odds ratio (OR) was the measure of effect size, with 95% confidence interval (CI) for each outcome. The total number of patients and those experiencing the event of interest in each group separately were extracted or computed from studies where such data were available to compute study-specific ORs. For the sensitivity, specificity, and predictive values, two by two contingency tables were extracted or calculated from the studies.

Pooled ORs based on raw data were calculated using the Mantel–Haenszel method [27,28]. To estimate the variance measure of heterogeneity (τ2), the Paule–Mandel method [29] was applied, alongside the Q profile method for the confidence interval. Details of the meta-analysis of sensitivity, specificity, and predictive values can be found in the Supplementary Material.

Statistical significance was determined by the absence of a null value within the confidence interval (CI). The meta-analysis findings were synthesized in forest plots. Between-study heterogeneity was elucidated using Higgins and Thompson’s I^2^ statistics [30]. All statistical analyses were conducted using the R software (R Core Team 2023, v4.2.3) [31] using the meta (Schwarzer 2023, v6.5.0) [32] and dmetar (Cuijpers, Furukawa, and Ebert 2023, v0.0.9000) [33] packages. Further details regarding calculations, data synthesis, publication bias assessment, and influential analyses can be found in the Supplementary Material.

### 2.10. Reporting Bias Assessment

Funnel plots were applied to report and visualize publication bias.

## 3. Results

### 3.1. Literature Search Results

**Figure 1:**
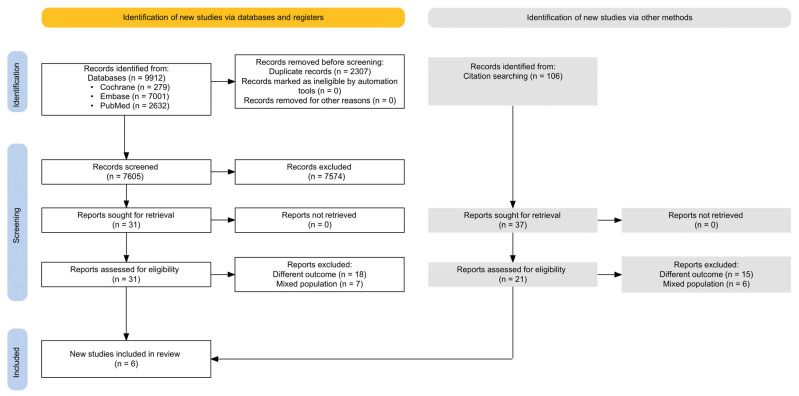
PRISMA 2020 flowchart representing the study search and selection process.

The search of the databases yielded 9912 initially relevant records. Ultimately, six eligible studies [15,34,35,36,37,38] involving 951 patients were included in the final analysis. Of these six studies, four studies [15,35,37,38] were included in the meta-analysis of hemorrhagic transformation, and three studies [15,34,36] in the meta-analysis for a less favorable 90-day functional outcome. The entire selection process is illustrated in the PRISMA diagram in Figure 1. Our calculation yielded a Cohen’s coefficient (κ) of 0.64 and 82.35% agreement for full-text selection.

### 3.2. Basic Characteristics of Included Studies

The final set of eligible studies was published between February 2012 and July 2021. The detailed characteristics of the included studies are presented in Table 1.

The number of patients with FLAIR-positive lesions was 246 (25.86% of the total number of patients). Four studies [15,34,35,38] were based on prospective cohorts, while two studies [36,37] were retrospective. MRI FLAIR status was generally interpreted by multiple examiners rather than a single examiner.

### 3.3. FLAIR Status and Hemorrhagic Transformation

Four of six identified studies [15,35,37,38] reported hemorrhagic transformation rates among 186 participants with FLAIR-positive and 618 participants with FLAIR-negative lesions. Across the studies involved, hemorrhagic transformation was defined as evidence of hemorrhage in the lesion at follow-up imaging 24 h after the IVT.

**Figure 2:**
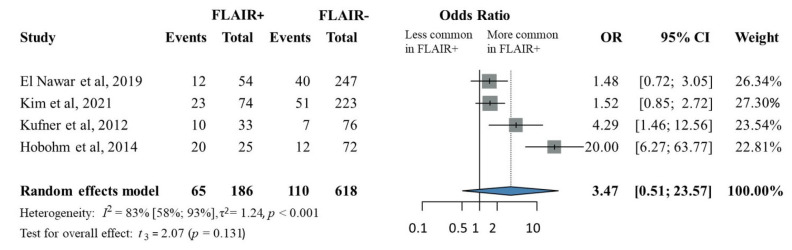
The odds ratio of hemorrhagic transformation in FLAIR-positive patients compared to FLAIR-negative patients. (FLAIR: fluid-attenuated inversion recovery) [15,35,37,38].

Patients with FLAIR-positive lesions who underwent IVT within the standard treatment window had a non-significantly higher odds of hemorrhagic transformation compared to the patients with FLAIR-negative lesions (OR, 3.47; 95% CI, 0.51–23.57; *p* = 0.131; Figure 2). There was substantial heterogeneity across the included studies (*p* for heterogeneity < 0.001; I^2^ = 83%).

We identified Hobohm et al. [37] as an outlier study in the hemorrhagic transformation analysis, primarily due to the inclusion of patients with more severe strokes, reflected by higher baseline NIHSS scores (mean 13.2 ± 7.1 overall; 14.9 ± 6.9 in the FLAIR-positive group). In a subgroup analysis restricted to patients with moderately severe strokes (median baseline NIHSS 6–10), the FLAIR-positive group showed a non-significantly increased risk of hemorrhagic transformation, albeit with a smaller effect size (OR 1.89, 95% CI 0.51–7.05, *p* = 0.174; Figure S1). Additional subgroup analyses assessing sensitivity, specificity, positive predictive value, and negative predictive value were also conducted (Figures S2 and S3).

**Figure 3:**
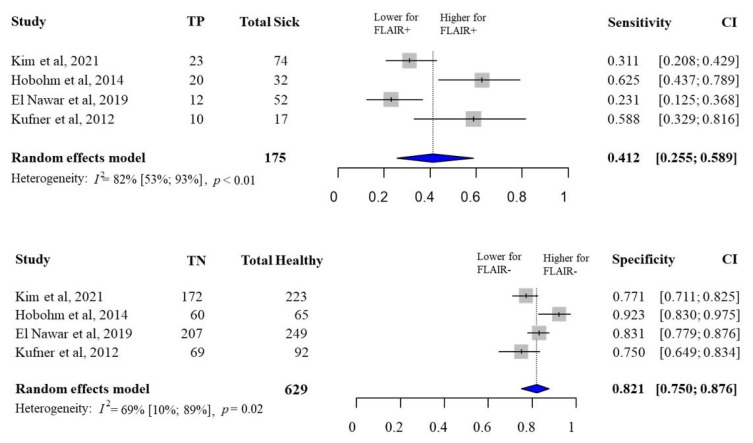
The pooled sensitivity and specificity of FLAIR status for hemorrhagic transformation. (FLAIR, fluid-attenuated inversion recovery) [15,35,37,38].

The pooled sensitivity of FLAIR-positive lesions for hemorrhagic transformations was 41.2% (95% CI, 25.5–58.9%), and the pooled specificity of FLAIR-negative lesions for hemorrhagic transformations was 82.1% (95% CI, 75–87.6%) (Figure 3). The pooled positive predictive value of FLAIR-positive lesions for hemorrhagic transformation was 39% (95% CI, 12–76%), and the pooled negative predictive value of FLAIR-negative lesions for hemorrhagic transformation was 83% (95% CI, 74–90%) (Figure S4).

### 3.4. FLAIR Status and Less Favorable 90-Day Functional Outcome

Three of six studies [15,34,36] reported 90-day functional outcome among 95 participants with FLAIR-positive and 310 participants with FLAIR-negative lesions.

A less favorable functional outcome was defined as a modified Rankin Scale (mRS) score of ≥ 2 by Kim et al. [15], and Emeriau et al. [36]. Ebinger et al. [34] defined this outcome as an mRS score > 2.

**Figure 4:**
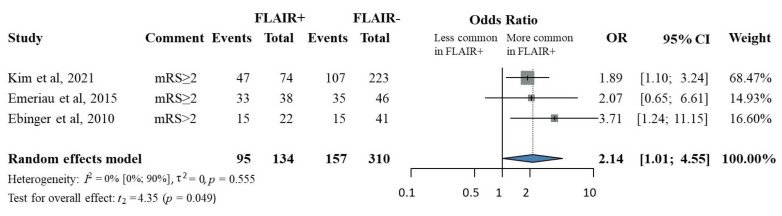
The odds ratio of a less favorable 90-day functional outcome in FLAIR-positive patients compared to FLAIR-negative patients. (FLAIR, fluid-attenuated inversion recovery) [15,34,36].

Patients with FLAIR-positive ischemic lesions who underwent IVT within the standard treatment window had significantly higher odds of having a less favorable 90-day functional outcome compared to patients with FLAIR-negative lesions (OR, 2.14; 95% CI, 1.01–4.55; *p* = 0.049; Figure 4). There was no significant heterogeneity among the included studies (*p* for heterogeneity = 0.555; I^2^ = 0%).

**Figure 5:**
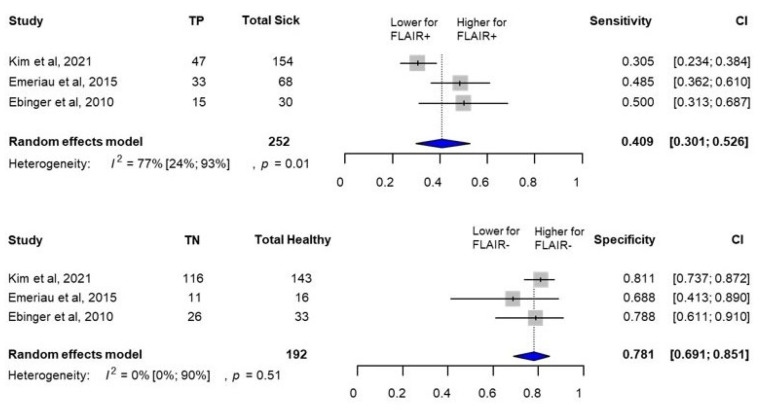
The pooled sensitivity and specificity of FLAIR status for less favorable 90-day functional outcome. (FLAIR, fluid-attenuated inversion recovery) [15,34,36].

The pooled sensitivity of FLAIR-positive lesions for less favorable 90-day functional outcome was 40.9% (95% CI, 30.1–52.6%), and the pooled specificity of FLAIR-negative lesions was 78.1% (95% CI, 69.1–85.1%) for less favorable 90-day functional outcome (Figure 5). The pooled positive predictive value of FLAIR-positive lesions for less favorable 90-day functional outcome was 73% (95% CI, 39–92%), and the pooled negative predictive value of FLAIR-negative lesions for less favorable 90-day functional outcome was 46% (95% CI, 13–82%) (Figure S5).

### 3.5. Risk of Bias Assessment

Most included studies carried a moderate risk of bias. Of the six studies included in the meta-analysis, two (33.3%) had a high risk of bias, and one study (16.6%) had a low risk of bias. Detailed results of the risk bias assessment are shown in Table S1 and are based on the QUIPS tool.

### 3.6. Publication Bias

**Figure 6:**
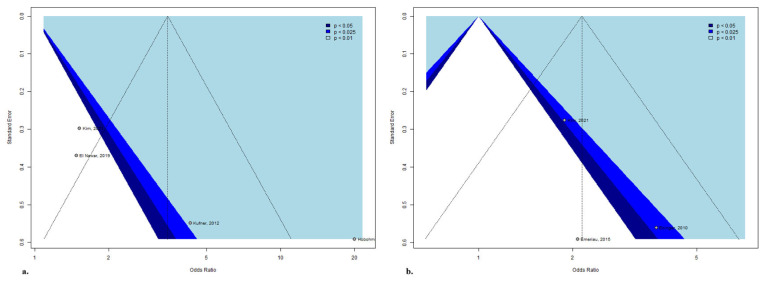
The funnel plot of the studies assessing (**a**) hemorrhagic transformation, (**b**) less favorable 90-day functional outcome [15,34,35,36,38].

Publication bias was examined by visual inspection of funnel plots, where the standard error was plotted against the difference in means. The asymmetry in the funnel plots suggested potential publication bias, but the interpretation is limited by the low number of studies involved in the meta-analysis (Figure 6. Influence measures and plots, such as leave-one-out analysis for changes in fitted values and L’Abbe and Bujat plots, were utilized to examine model fitting parameters and identify potential outlier publications (Figures S6–S10).

### 3.7. Quality of Evidence

As we included only cohort studies, the certainty of evidence was low for both outcomes. Detailed results of the GRADE assessment can be found in Table S2.

## 4. Discussion

This study aimed to investigate the association between FLAIR status and the outcomes after IVT in known-onset strokes. Therefore, we analyzed the rates of hemorrhagic transformation and 90-day functional outcome after reperfusion treatment. Our analysis revealed higher rates of hemorrhagic transformation and less favorable 90-day functional outcome after treatment in patients with FLAIR-positive acute ischemic lesions than in patients with FLAIR-negative lesions. We found a statistically significant difference between the two groups for 90-day functional outcome and a non-significant difference for hemorrhagic transformation. Moreover, we found that more than a quarter of patients presenting within 4.5 h had FLAIR-positive lesions, indicating that FLAIR positivity did not rule out recent strokes.

The diagnostic role of FLAIR has been intensely utilized in the context of wake-up strokes [39]. A DWI-FLAIR mismatch occurs when there is a hyperintense signal on DWI without a corresponding lesion on FLAIR, suggesting that ischemic changes have occurred recently, and the patient is likely to be within a time window for safe and effective thrombolysis [39,40,41]. Although our analysis found an association between FLAIR-positive acute ischemic lesions and worse functional outcome, the prognostic value of FLAIR imaging in this context remains uncertain. In particular, the modest sensitivity observed suggests that FLAIR status alone is unlikely to serve as a reliable predictor of outcome. Therefore, FLAIR-positivity in early known-onset strokes may prompt a more careful risk-benefit assessment but should not be considered a reason to withhold treatment in otherwise eligible patients.

Our findings are in line with those of a previous meta-analysis, which showed that FLAIR-positive acute ischemic lesions due to impaired blood–brain barrier are associated with a more severe ischemic stroke and increased likelihood of vessel-wall failure leading to hemorrhagic transformation and neurological deterioration [42,43]. Further studies confirmed that FLAIR-negative acute ischemic lesions were more likely to show early neurological improvement than FLAIR-positive lesions [44]. Radiomics based on FLAIR and other imaging sequences have shown good performance in predicting functional outcome after acute ischemic stroke [45,46]. Furthermore, recent findings show that synthetic FLAIR generated from DWI using deep learning offers diagnostic performance comparable to real FLAIR in detecting early ischemic stroke, enabling a clinically meaningful reduction of approximately 25% in MRI protocol duration [47,48].

Several prognostic scores, such as HAT, DRAGON, Stroke-TPI, SPAN-100, MSS, SEDAN, SITS-ICH, iScore, and ASTRAL, have been used to forecast the likelihood of hemorrhagic transformation following IVT [49]. Three of them (DRAGON, HAT, and SEDAN) incorporate CT-based radiological variables as major components [49]. Additionally, CT perfusion has shown high sensitivity and moderately high specificity in predicting hemorrhagic transformation in acute ischemic stroke [50]. Notably, none of these prognostic models considers MRI variables as principal prognostic factors for hemorrhagic transformation after IVT [49].

### 4.1. Strengths and Limitations

Our analysis strictly followed a pre-registered protocol, ensuring transparency and minimizing bias in our investigation of FLAIR positivity and post-IVT outcomes. The strength of our study lies in the conduction of univariate analysis, which provides a comprehensive and objective assessment of the predictive value of FLAIR positivity. A consistent and clear definition of FLAIR positivity across studies ensures uniformity and clarity in our analysis, increasing the reliability of our conclusions.

However, several limitations should be taken into account. One limitation of our study is the relatively low number of studies and patients involved, which potentially limited the generalizability of our findings on the predictive value of FLAIR positivity. Moreover, the majority of enrolled patients suffered moderate strokes based on the reported baseline NIHSS scores, restricting the applicability of our results to more severe stroke populations. However, in the one study with more severe strokes (Hobohm et al.) [37], the odds for hemorrhagic transformation in FLAIR-positive patients were much higher than in the other studies with milder strokes, so we expect this association to become stronger with increasing stroke severity. In mild-to-moderate acute ischemic stroke, the prognostic impact of hemorrhagic transformation may be less pronounced than in severe cases. Nevertheless, hemorrhagic transformation remains clinically relevant, as it can lead to secondary neurological worsening, delay functional recovery, and influence post-thrombolytic management decisions. This relevance is further underscored by the recently introduced concept of the “leaky core,” which describes regions within the ischemic core where irreversible neuronal injury coincides with severe blood–brain barrier disruption, predisposing to hemorrhagic transformation and malignant edema after reperfusion [51]. We also acknowledge that using hemorrhagic transformation rather than symptomatic intracerebral hemorrhage (sICH) as an outcome measure reduces direct clinical applicability. However, as most included studies did not consistently distinguish between symptomatic and asymptomatic hemorrhagic events, hemorrhagic transformation was used as the most uniformly reported endpoint. Despite this limitation, hemorrhagic transformation remains a relevant surrogate marker of tissue vulnerability and provides insight into the imaging–pathophysiological relationship between FLAIR positivity and secondary injury after reperfusion therapy. The short duration of follow-up in the included studies may hinder our ability to assess the long-term predictive value of FLAIR positivity accurately. Additionally, variations in FLAIR positivity and hemorrhagic transformation screening techniques across articles may cause heterogeneity, affecting the comparability of results. Finally, the presence of a moderate to high risk of bias in some domains of the included studies could potentially affect the validity of our conclusions about the predictive role of FLAIR positivity in stroke outcomes after thrombolysis. While FLAIR negativity has demonstrated good positive predictive value for identifying strokes within the first 4.5 h, FLAIR positivity has poor negative predictive value. Consequently, a DWI–FLAIR match does not reliably exclude early presentation. Interpretation of FLAIR hyperintensity can also be challenging in the presence of chronic white matter hyperintensities of vascular origin, and inter-observer variability remains an inherent limitation.

Taken together, these considerations underscore that while FLAIR remains a valuable imaging marker of ischemic tissue evolution, its interpretation requires contextualization within the clinical and imaging profile of each patient.

### 4.2. Implications for Clinical Practice and Future Research

While our findings show a significant association between FLAIR hyperintensity and less favorable functional outcome and a non-significantly higher odds for hemorrhagic transformation post-IVT, the clinical relevance of FLAIR status remains uncertain. Given the limited number of studies, high between-study heterogeneity, and the modest effect sizes, these findings should be interpreted with caution. Based on current evidence, FLAIR positivity should not be used as a standalone decision-making tool, and its presence should not be a reason to withhold treatment from otherwise eligible patients. Integrating FLAIR positivity assessment into clinical practice may improve prognostication and inform post-thrombolysis treatment and follow-up, e.g., timing of follow-up scans, timing of secondary prevention antithrombotic use and intensity of clinical observation. The study highlights the need for further research to validate and refine the predictive role of FLAIR positivity in stroke outcomes following IVT. Future studies could focus on prospective investigations to confirm the findings of this meta-analysis and elucidate the underlying mechanisms that drive the association between FLAIR positivity and hemorrhagic transformation, possibly with the addition of perfusion data. Adjusted analyses are needed to determine whether FLAIR positivity has independent predictive value when accounting for other relevant clinical and imaging factors. Additionally, research should assess the impact of FLAIR positivity on long-term recovery and its utility in post-thrombolysis management. The potential of artificial intelligence and radiomics to enhance the diagnostic and predictive value of FLAIR and other MRI markers should be explored. External validation of prognostic models, including FLAIR status, is also recommended. Overall, these findings should be interpreted with caution and viewed as a basis for further investigation rather than as definitive evidence for the clinical use of FLAIR positivity in acute stroke care. Understanding scientific principles and knowledge is essential for translating and integrating scientific discoveries to improve healthcare [52,53].

## 5. Conclusions

In conclusion, our study shows that FLAIR positivity in acute ischemic stroke treated with IVT within the standard time window is associated with less favorable functional outcome and a trend toward higher hemorrhagic transformation risk. FLAIR positivity may be taken into account in treatment decisions and patient management, but should not be regarded as a contraindication to IVT in otherwise eligible patients. Further prospective research is needed on the clinical significance of FLAIR positivity and its underlying mechanisms.

## Figures and Tables

**Table 1:** Basic characteristics of included studies.

Author (Year)	Study Site	Study Design	No. of Patients (Female)	Age (Year) ‡	Outcome	MRI SMF	MRI Protocol	Follow-Up Imaging	NIHSS FLAIR+ ‡	NIHSS FLAIR- ‡	LKN-to-IVT FLAIR+ (Min) ‡	LKN-to-IVT FLAIR− (Min) ‡
Ebinger 2012 [34]	Germany	Prospective cohort	44 (48.88%)	72.0 (65–81)	90-day functional outcome	ND	DWI, FLAIR, MRA	NA	NA	5 (3.5–8)	ND	130 (108–154)
Kufner 2012 [38]	Germany	Prospective cohort	60 (55.04%)	71.3 (±12.5)	HT	1.5/3T	DWI, FLAIR, T2* (PWI, MRA-optional)	MRI	10 (6–17)	6 (4–11)	120 (95–165)	124 (98–152)
Hobohm 2014 [37]	Germany	Retrospective cohort	42 (43.29%)	70.7 (±11.7)	HT	1.5T	DWI, FLAIR, T2*, PWI, ToF MRA	CT	14.9 (±6.9)	12.7 (±7.1)	ND	ND
Emeriau 2015 [36]	France	Retrospective cohort	36 (42.85%)	64.0 (50–75)	90-day functional outcome	3T	DWI, FLAIR	NA	14 (10–20)	14 (9–19)	190 (160–201)	162 (140–190)
El Nawar 2019 [35]	France	Prospective cohort	145 (48.17%)	71.3 (±15.9)	HT	1.5T	DWI, FLAIR, T2*, ADC, ToF MRA	CT/MRI	ND	ND	ND	ND
Kim 2021 [15]	USA	Prospective cohort	143 (48.14%)	70.0 (59–83)	HT; 90-day functional outcome	1.5T	DWI, FLAIR, ADC, GRE, MRA (post-contrast FLAIR)	MRI	7 (4–13)	6 (3–15)	169 (120–208)	142 (107–188)

‡ Parameters represented as mean with standard deviation, or median with range (minimum and maximum); (ADC, apparent diffusion coefficient; CT, computed tomography; DWI, diffusion-weighted imaging; FLAIR, fluid-attenuated inversion recovery; GRE, gradient-recalled echo; HT, hemorrhagic transformation; IVT, intravenous thrombolysis; LKN, last known normal; MRA, magnetic resonance angiography; MRI, magnetic resonance imaging; NA, not applicable; ND, not defined; NIHSS, National Institute of Health Stroke Scale; PWI, perfusion-weighted imaging; SMF: strength of magnetic field; T: tesla; T2*, T2-star; ToF MRA, time-of-flight magnetic resonance angiography).

## Data Availability

All data generated and analyzed during this study are included in this article, the Supplementary Material files, and in the full-text articles included in this meta-analysis. Further inquiries can be directed to the corresponding author (B.G.).

## Supplementary Materials

The following supporting information can be downloaded at https://www.mdpi.com/article/10.3390/jcm14228031/s1: Supplementary Text, Figures S1–S3: Subgroup analysis; Figure S4: The pooled positive and negative predictive values of FLAIR status for hemorrhagic transformation; Figure S5: The pooled positive and negative predictive values of FLAIR status for less favorable 90-day functional outcome; Figure S6: L’Abbe plots; Figure S7: Baujat plots; Figure S8: Leave-one-out analysis; Figure S9: Influence diagnostics; Figure S10: Influence forest plots; Table S1: Risk of bias assessment using Quality in Prognostic Studies (QUIPS) tool; Table S2: Grading of Recommendations, Assessment, Development and Evaluations (GRADE) assessment.
