# Supplementary material for: Association of FLAIR Positivity and Worse Outcomes After Intravenous Thrombolysis in Known-Onset Strokes: A Systematic Review and Meta-Analysis
Source: J Clin Med. 2025 Nov 12;14(22):8031. doi: 10.3390/jcm14228031 (PMC12653928; doi:10.3390/jcm14228031)
Supplement: Supplementary file 1 [file jcm-14-08031-s001.zip › jcm-3940430-supplementary.pdf]

SUPPLEMENTARY ONLINE MATERIAL

Association of FLAIR positivity and worse outcomes after intravenous thrombolysis in known-onset strokes: a systematic review and meta-analysis

Summary:

1. **Supplementary Methods**

2. **Supplementary Figures**

   Figure S1. Subgroup analysis

   Figure S2. Subgroup analysis

   Figure S3. Subgroup analysis

   Figure S4. The pooled positive and negative predictive values of FLAIR status for hemorrhagic transformation. (FLAIR, fluid-attenuated inversion recovery)

   Figure S5. The pooled positive and negative predictive values of FLAIR status for less favorable 90-day functional outcome. (FLAIR, fluid-attenuated inversion recovery)

   Figure S6. L'Abbe plots for a) hemorrhagic transformation b) less favorable 90-day functional outcome.

   Figure S7. Baujat plots for a) hemorrhagic transformation b) less favorable 90-day functional outcome.

   Figure S8. Leave-one-out analysis a) hemorrhagic transformation b) less favorable 90-day functional outcome.

   Figure S9. Influence diagnostics for a) hemorrhagic transformation b) less favorable 90-day functional outcome.

   Figure S10. Influence forest plots for a) hemorrhagic transformation b) less favorable 90-day functional outcome.

3. **Supplementary Tables**

   Table S1. Risk of bias assessment using Quality in Prognostic Studies (QUIPS) tool.

   Table S2. Grading of Recommendations, Assessment, Development and Evaluations (GRADE) assessment.

Supplementary Methods

1.1. Meta-analysis of odds ratios

In cases of zero cell counts, the exact Mantel-Haenszel method (without continuity correction) was applied. The Hartung-Knapp adjustment was used.

1.2. Meta-analysis of diagnostic outcomes

To pool sensitivity and specificity, the bivariate model of (Reitsma et al. 2005; Chu and Cole 2006) was fitted using the R script of the online tool described by Freeman (2019). This approach takes into account the dependency between sensitivity and specificity. The result is a confidence region containing pooled sensitivity and 1-specificity in 95% of the cases. The analysis was repeated for the subgroup as well as with all four studies, and the pooled estimates and CI derived from the confidence regions are visualized on forest plots. Heterogeneity was assessed by performing separate univariate analyses of sensitivity, and specificity using the generalized mixed-effect approach of Stijnen, Hamza, and Özdemir et al. and calculating the I^2 measure and its confidence interval. The same approach was used to pool predictive values. Note that predictive values depend on the prevalence of the condition being predicted. In the included studies, this varied moderately for hemorrhagic transformation and considerably for the functional outcome. Publication bias could not be meaningfully assessed due to the low number of studies.

1.3. Additional meta-analytic methods (irrespective of effect size measure)

Assessment of small-study publication bias involved visual examination of Funnel plots and computation of the p-value from Egger's test. We posited the presence of potential small study bias if the p-value was below 10%. However, it was acknowledged that the diagnostic capability of the test was limited with fewer than approximately 10 studies. Identification of potential outlier publications entailed exploring various influence measures and plots in accordance with the recommendations of Harrer et al. Different influence measures and plots, such as leave-one-out analysis for changes in fitted values and Bujat diagnostics values and plots, were utilized to examine model fitting parameters and potential outlier publications.

Supplementary Figures

Figure S1. Subgroup analysis

Legend: Forest plots representing the odds of hemorrhagic transformation in studies of moderate strokes with a median baseline NIHSS score from 6 to 10, and moderate strokes with a mean baseline NIHSS score of 13.2. (NIHSS, National Institute of Health Stroke Scale)

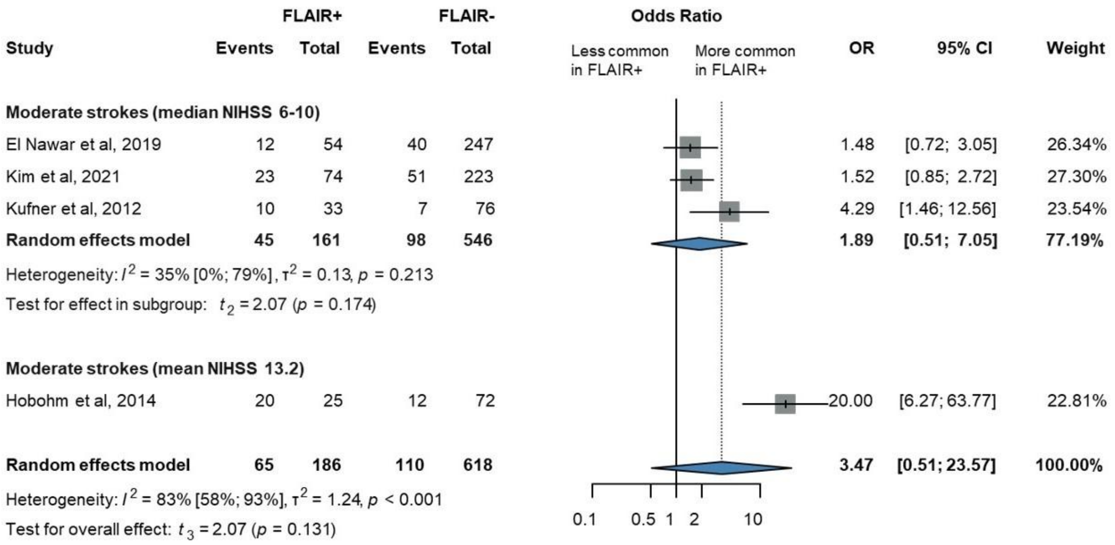

Figure S2. Subgroup analysis

Legend: Forest plot representing the pooled sensitivity and specificity of FLAIR status for hemorrhagic transformation in studies of moderate strokes with a median baseline NIHSS score from 6 to 10, and moderate strokes with a mean baseline NIHSS score of 13.2. (FLAIR, Fluid-Attenuated Inversion Recovery; NIHSS, National Institute of Health Stroke Scale)

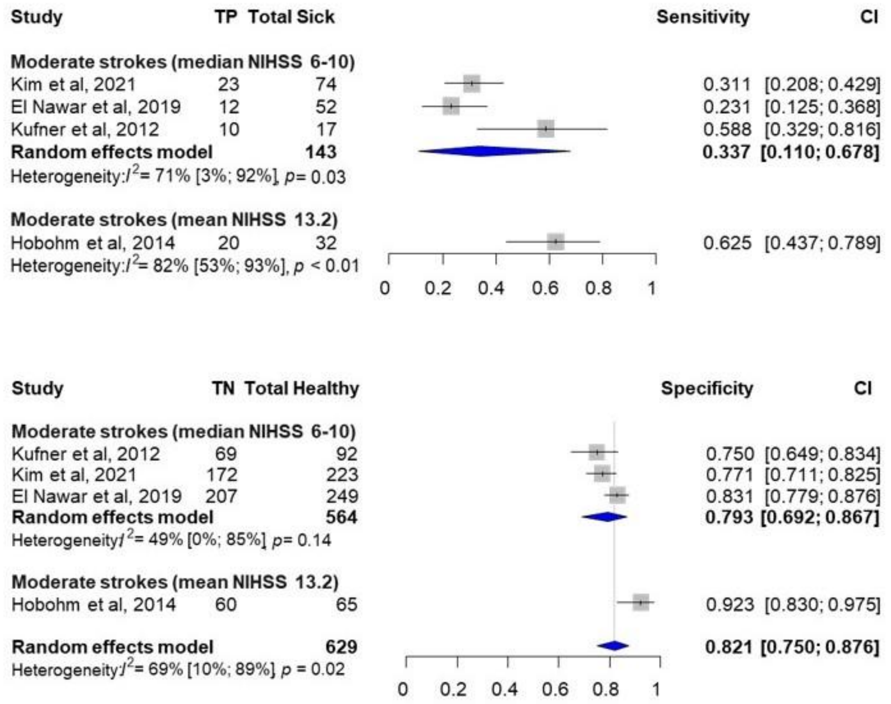

Figure S3. Subgroup analysis

Legend: Forest plot representing the pooled positive and negative predictive values of FLAIR status for hemorrhagic transformation in studies of moderate strokes with a median baseline NIHSS score from 6 to 10, and moderate strokes with a mean baseline NIHSS score of 13.2. (FLAIR, Fluid-Attenuated Inversion Recovery; NIHSS, National Institute of Health Stroke Scale)

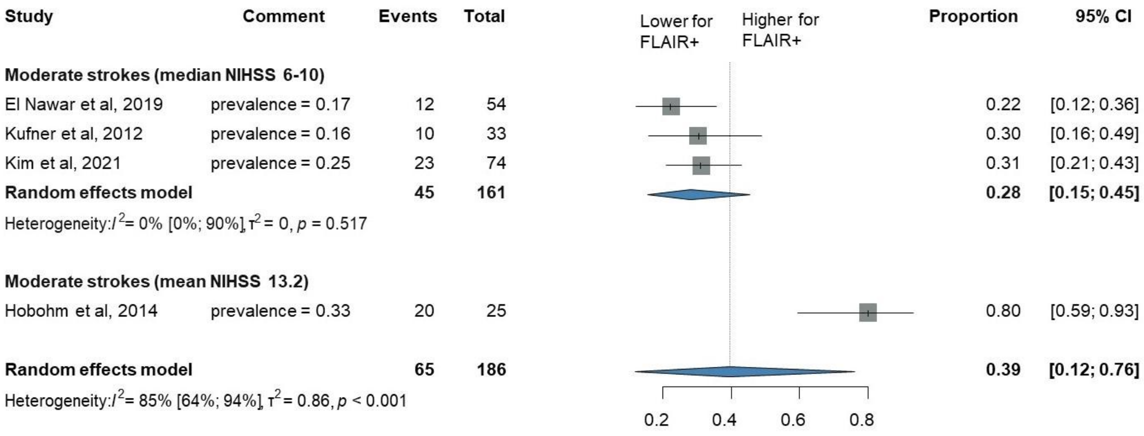

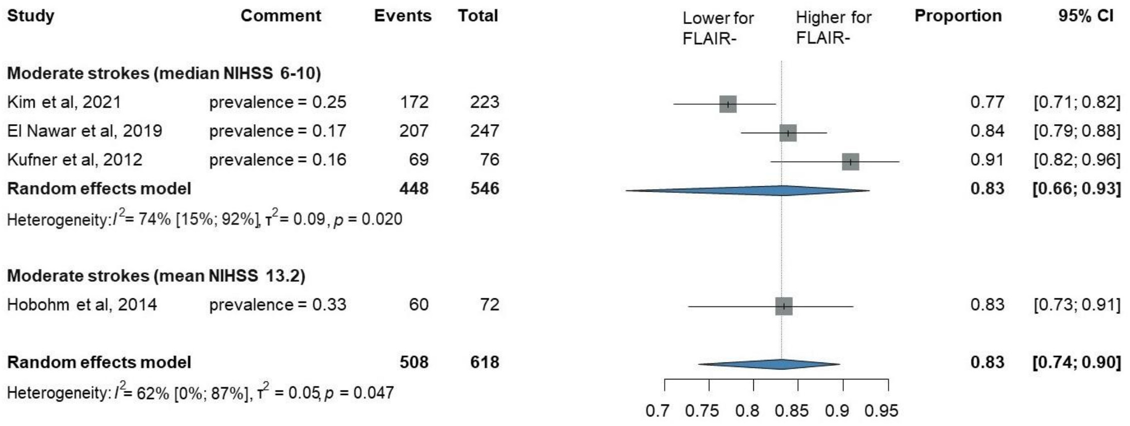

Figure S4. Forest plot representing the pooled positive and negative predictive values of FLAIR status for hemorrhagic transformation. (FLAIR, fluid-attenuated inversion recovery)

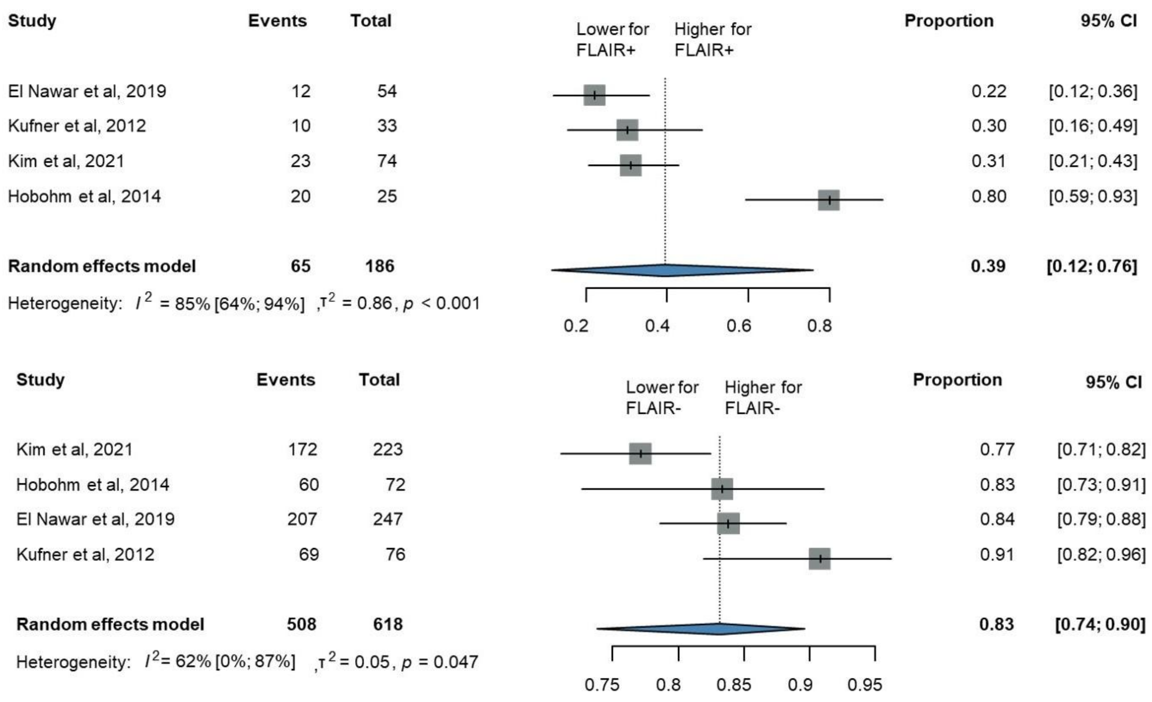

Figure S5. Forest plot representing the pooled positive and negative predictive values of FLAIR status for less favorable 90-day functional outcome. (FLAIR, fluid-attenuated inversion recovery)

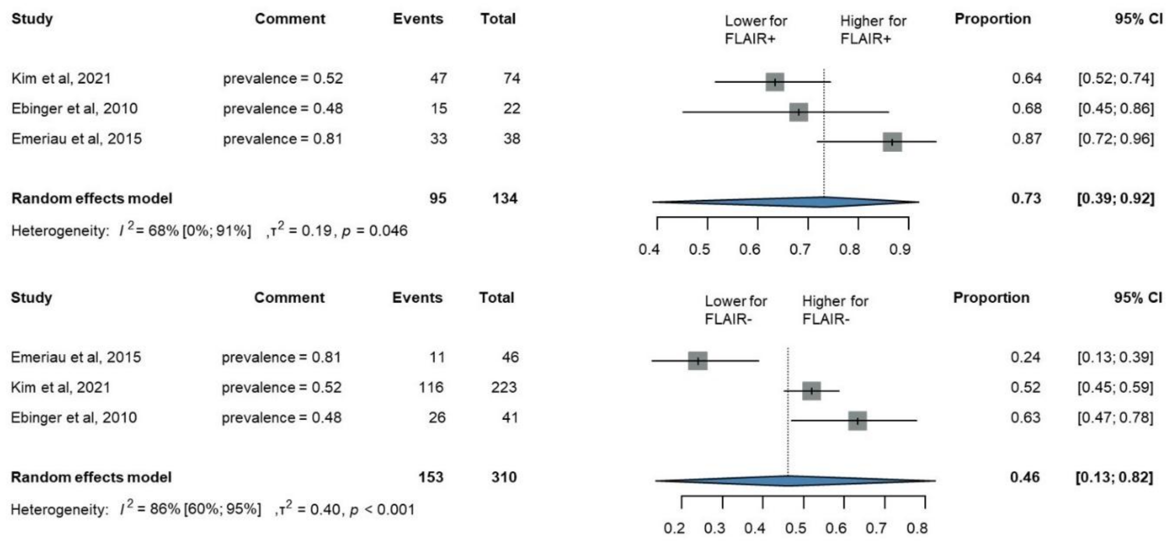

Figure S6. L'Abbe plots for a) hemorrhagic transformation b) less favorable 90-day functional outcome.

a)

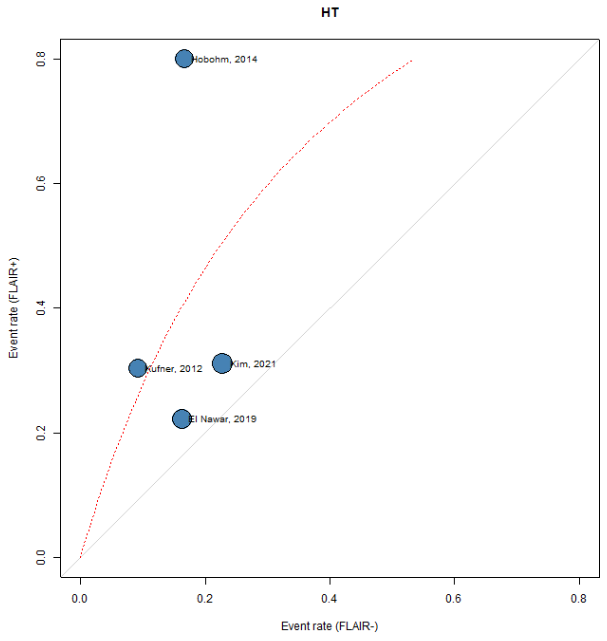

b)

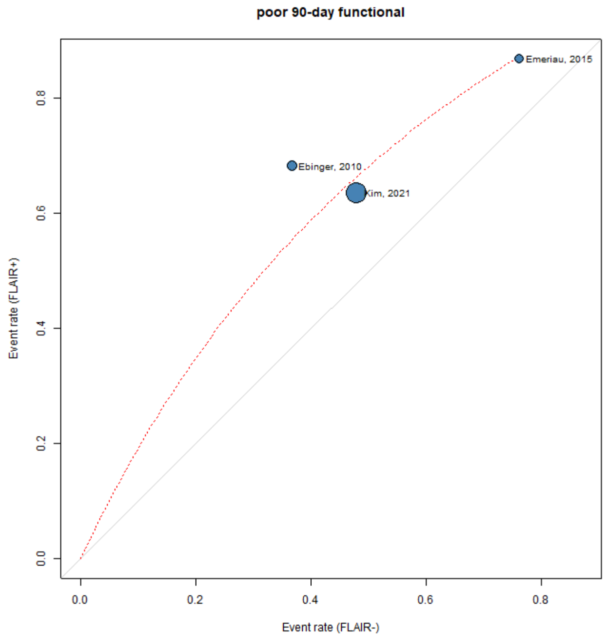

Figure S7. Baujat plots for a) hemorrhagic transformation b) less favorable 90-day functional outcome.

a)

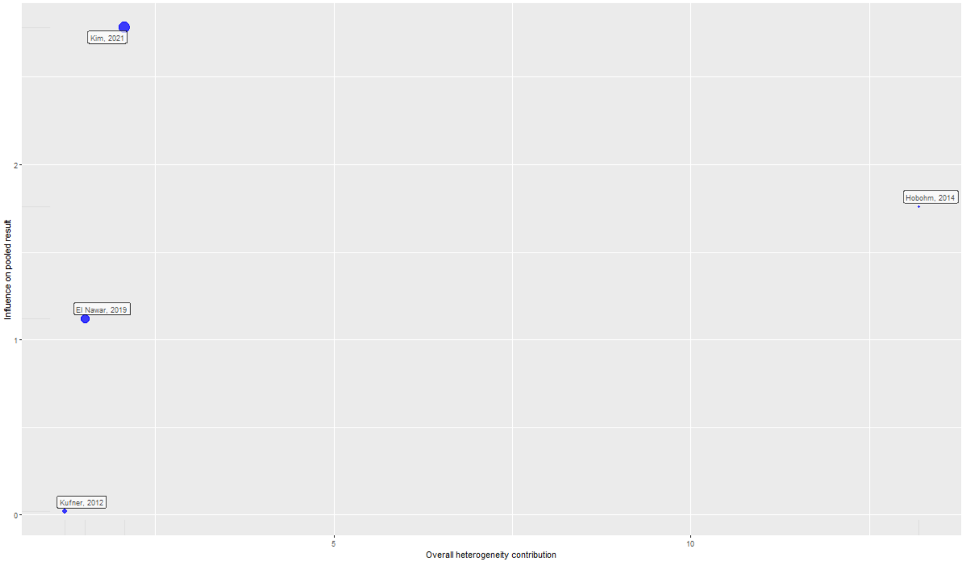

b)

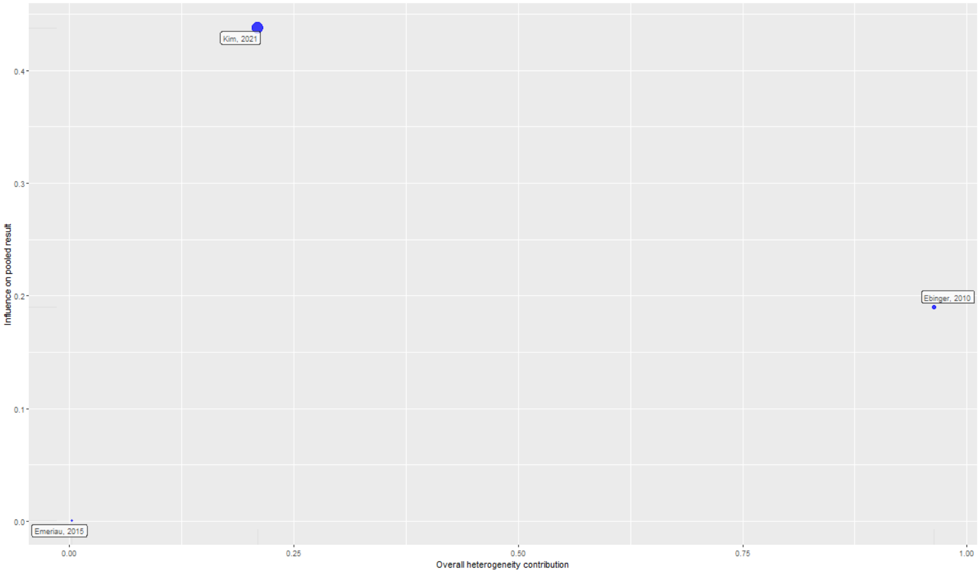

Figure S8. Leave-one-out analysis a) hemorrhagic transformation b) less favorable 90-day functional outcome.

a)

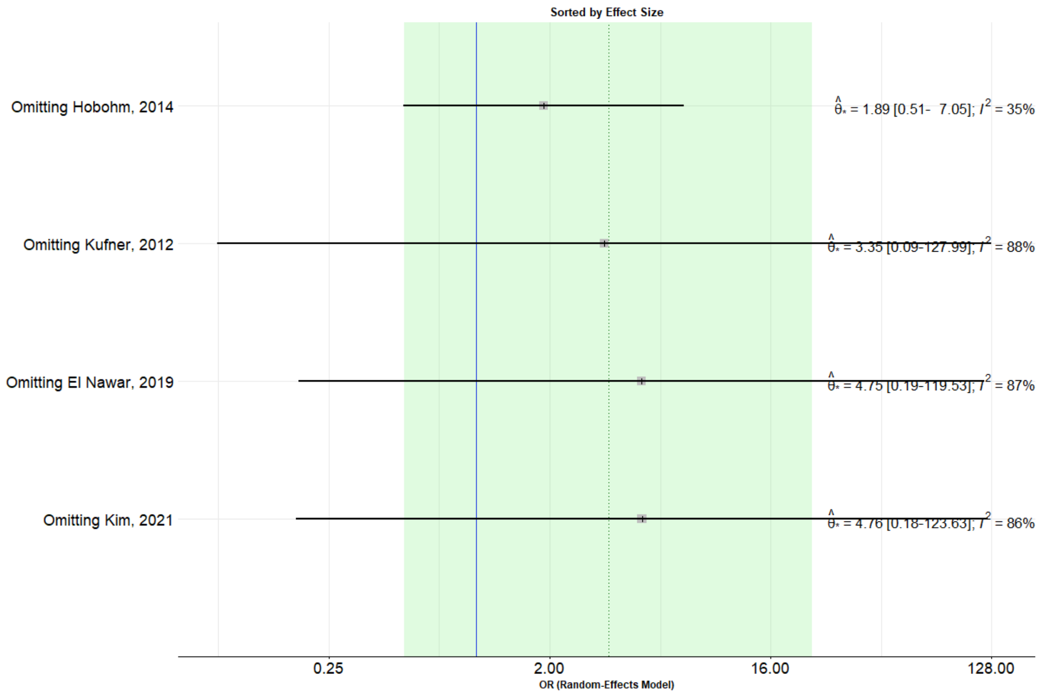

b)

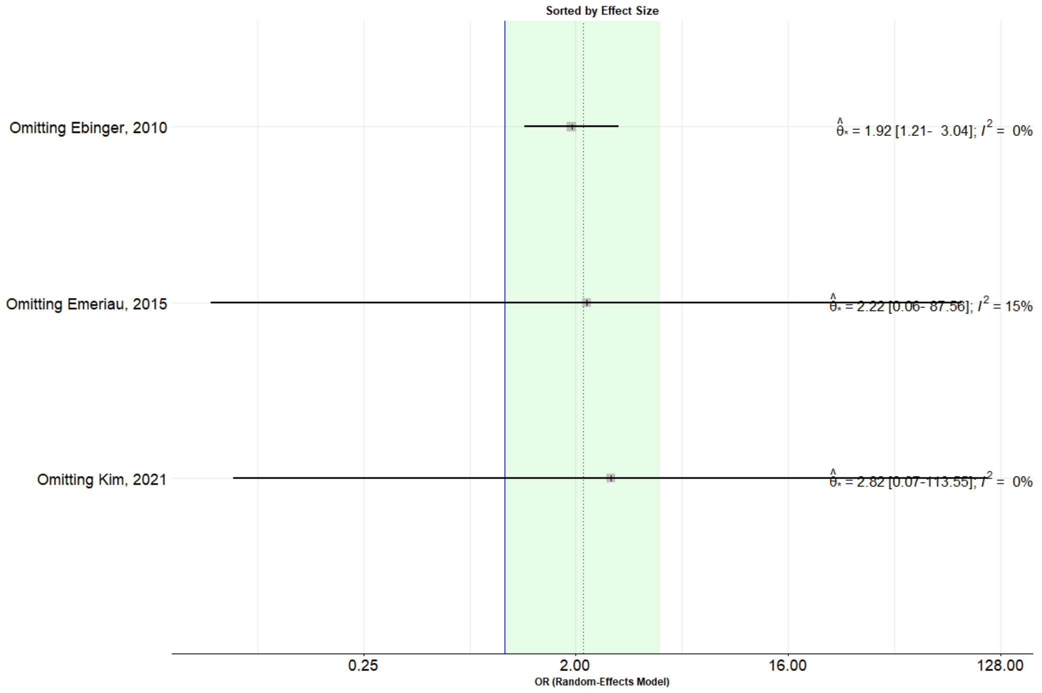

Figure S9. Influence diagnostics for a) hemorrhagic transformation b) less favorable 90-day functional outcome.

a)

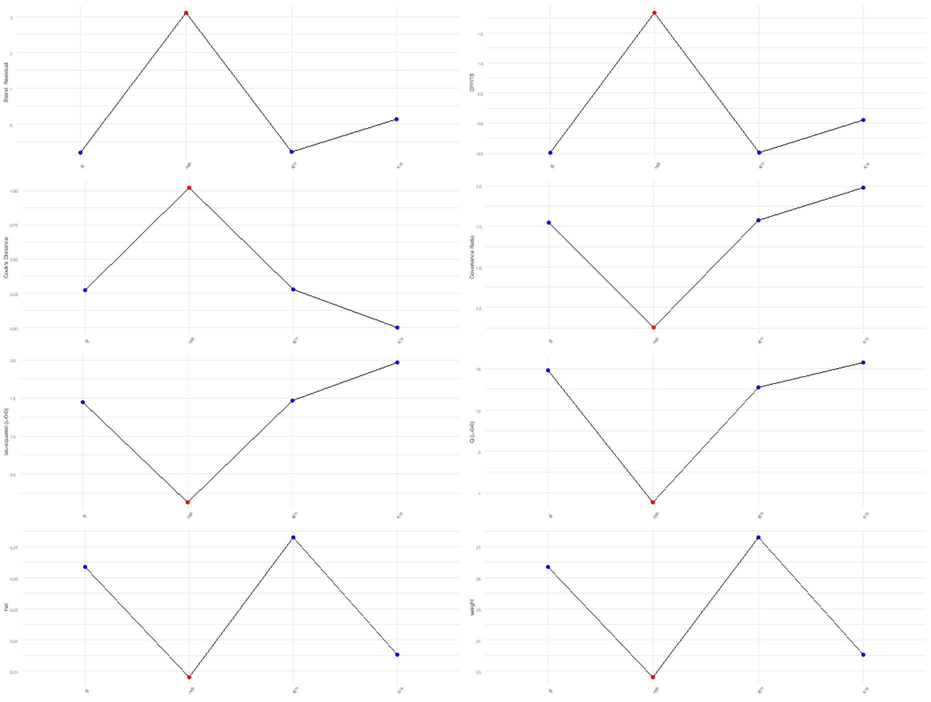

b)

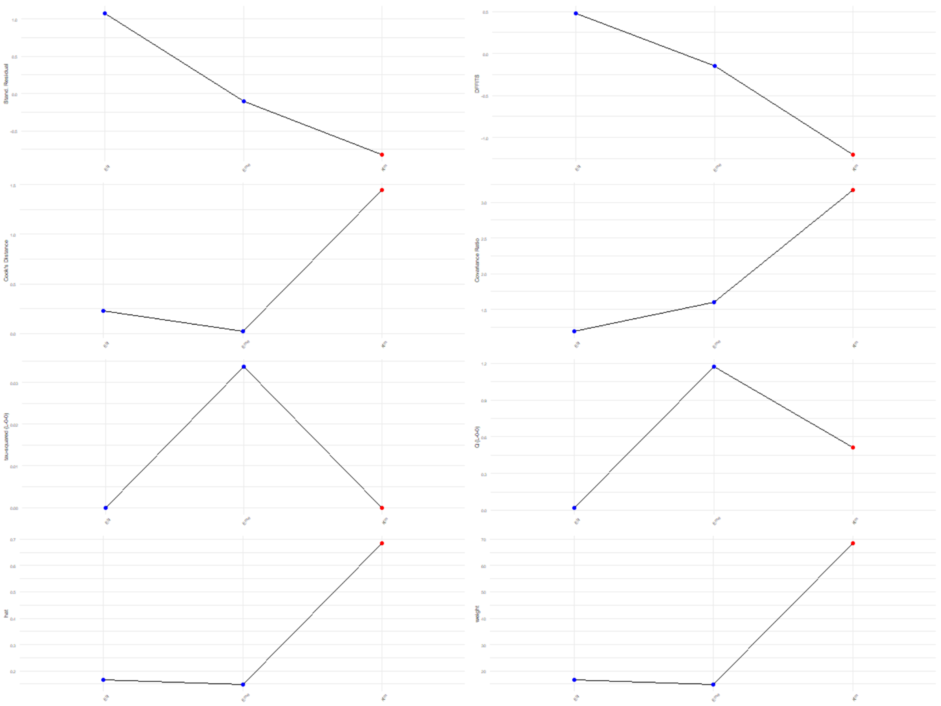

Figure S10. Influence forest plots for a) hemorrhagic transformation b) less favorable 90-day functional outcome.

a)

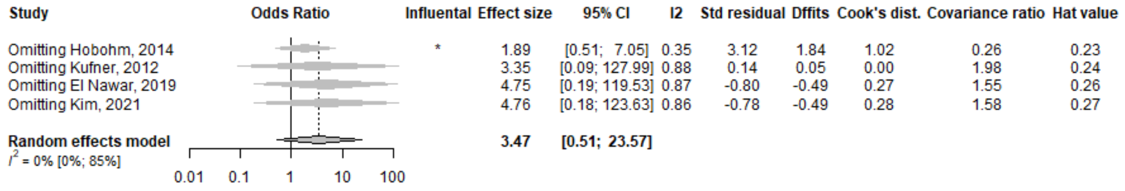

b)

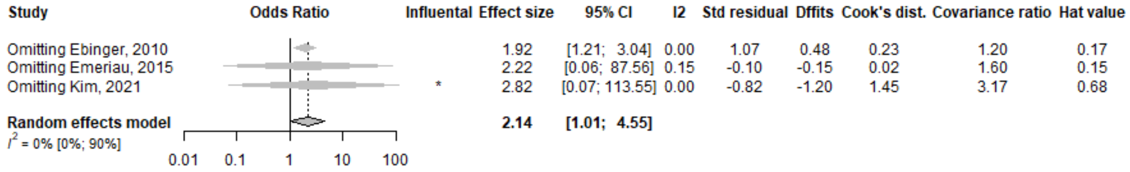

Supplementary Tables

Table S1. Risk of bias assessment using Quality in Prognostic Studies (QUIPS) tool for a) hemorrhagic transformation; b) less favorable functional outcome.

a)

Study	Study participation	Study attrition	Prognostic factor measurement	Outcome measurement	Study confounding	Statistical analysis reporting	Overall risk of bias
El Nawar et al. (2019)	?	+	+	+	+	+	?
Hobohm et al. (2014)	+	?	+	+	?	+	?
Kim et al. (2021)	+	?	+	+	?	+	?
Kufner et al. (2012)	?	-	+	+	+	+	-

b)

Study	Study participation	Study attrition	Prognostic factor measurement	Outcome measurement	Study confounding	Statistical analysis reporting	Overall risk of bias
Ebinger et al. (2012)	-	?	+	+	?	?	-
Emeriau et al. (2015)	+	+	+	+	+	+	+
Kim et al. (2021)	+	?	+	+	?	+	?

Table S2. Grading of Recommendations Assessment, Development and Evaluation (GRADE) assessment

Certainty assessment							№ of patients		Effect		Certainty	Importance
**№ of studies**	**Study design**	**Risk of bias**	**Inconsistency**	**Indirectness**	**Imprecision**	**Other considerations**	**FLAIR +**	**FLAIR -**	**Relative (95% CI)**	**Absolute (95% CI)**		
**Hemorrhagic transformation (assessed with: yes/no)**												
4	non-randomized studies	not serious	serious[a]	not serious[b]	not serious[c]	strong association	65/186 (34.9%)	110/618 (17.8%)	**OR 3.47** (0.51 to 23.57)	**251 more per 1,000** (from 79 fewer to 658 more)	⊕⊕◯◯ Low	IMPORTANT
**Less favorable 90-day functional outcome (follow-up: 90 days; assessed with: mRS)**												
3	non-randomized studies	not serious	serious[a]	not serious[b]	not serious[c]	strong association	95/134 (70.9%)	157/310 (50.6%)	**OR 2.14** (1.01 to 4.55)	**181 more per 1,000** (from 2 more to 317 more)	⊕⊕◯◯ Low	IMPORTANT

CI: confidence interval; FLAIR: fluid-attenuated inversion recovery; **OR:** odds ratio

Explanations

a. Difference in effect estimate and high heterogeneity.

b. Included participants had homogenous characteristics.

c. Did not cross benefit/harm line and 0-effect line.
